# Intraoperative radiotherapy for early breast cancer: do health professionals choose convenience or risk?

**DOI:** 10.1186/1748-717X-9-33

**Published:** 2014-01-25

**Authors:** Tammy Corica, David Joseph, Christobel Saunders, Max Bulsara, Anna K Nowak

**Affiliations:** 1 University of Western Australia PhD Candidate, School of Medicine and Pharmacology, Comprehensive Cancer Centre, Sir Charles Gairdner Hospital, Nedlands, WA 6009, Australia; 2 Radiation Oncology Clinical Trials and Research Unit, Comprehensive Cancer Centre, Sir Charles Gairdner Hospital, Nedlands, WA 6009, Australia; 3 School of Surgery, University of Western Australia, Comprehensive Cancer Centre, Sir Charles Gairdner Hospital, Nedlands, WA 6009, Australia; 4 Radiation Oncology, Comprehensive Cancer Centre, Sir Charles Gairdner Hospital, Nedlands, WA 6009, Australia; 5 School of Surgery, University of Western Australia, MBDP M507, Crawley, WA 6009, Australia; 6 Institute of Health and Research, University of Notre Dame, 19 Mouat Street, P.O Box 1225, Fremantle, WA 6959, Australia; 7 School of Medicine and Pharmacology, University of Western Australia, MBDP M503, Crawley, WA 6009, Australia

**Keywords:** Physician survey, Treatment preference, Patient preferences, Breast cancer, Intraoperative radiotherapy, Partial breast irradiation, TARGIT, Preference questionnaire, IORT, PBI

## Abstract

**Background:**

The randomized TARGIT trial comparing experimental intra-operative radiotherapy (IORT) to up to 7 weeks of daily conventional external beam radiotherapy (EBRT) recruited participants in Western Australia between 2003 and 2012. We aimed to understand preferences for this evolving radiotherapy treatment for early breast cancer (EBC) in health professionals, and how they changed over time and in response to emerging data. Preferences for single dose IORT or EBRT for EBC were elicited in 2004 and 2011, together with factors that may be associated with these preferences.

**Methods:**

Western Australian health professionals working with breast cancer patients were invited to complete a validated, self-administered questionnaire. The questionnaire used hypothetical scenarios and trade-off methodology to determine the maximum increase in risk of local recurrence health professionals were willing to accept in order to have a single dose of IORT in the place of EBRT if they were faced with this decision themselves.

**Results:**

Health professional characteristics were similar across the two time points although 2011 included a higher number of nurse (49% vs. 36%) and allied health (10% vs. 4%) participants and a lower number of radiation therapists (17% vs. 32% ) compared to 2004.

Health professional preferences varied, with 7.5% and 3% judging IORT unacceptable at any risk, 18% and 21% judging IORT acceptable only if offering an equivalent risk, 56% and 59% judging IORT acceptable with a low maximum increase in risk (1-3%) and 19% and 17% judging a high maximum increase in risk acceptable (4-5%), in 2004 and 2011 respectively. A significantly greater number of nurses accepted IORT as a treatment option in 2011.

**Conclusions:**

Most Western Australian health professionals working with breast cancer patients are willing to accept an increase in risk of local recurrence in order to replace EBRT with IORT in a hypothetical setting. This finding was consistent over two time points spanning 7 years despite the duration of clinical experience with IORT and the publication of the early clinical results of IORT in 2010. These results need to be compared with preferences elicited from patient groups, and further investigation into the impact of personal preferences on health professionals’ advice to patients is warranted.

## Background

Breast cancer is the most common cancer, and the second most common cause of cancer mortality after lung cancer in Australian women [1]. Whole breast external beam radiotherapy (EBRT) delivered in 25–35 daily fractions over a period of 6–7 weeks is standard adjuvant treatment for women undergoing breast conserving surgery for early breast cancer [2]–[6]. EBRT must be delivered in a radiation oncology facility, and may require temporary relocation for women who are geographically isolated or unable to travel daily. It is also inconvenient for those with family or work commitments. In addition, EBRT has potential immediate toxicities such as erythema, oedema and induration of the breast and can result in skin breakdown severe enough to require daily dressings. Potential long-term side effects include pulmonary fibrosis, cardiotoxicity, osteoradionecrosis, and the induction of secondary malignancies.

The recent development of Intra-Operative Radiotherapy (IORT) has made the delivery of radiation directly to the tissues at the site of the primary tumour possible, during a single session either at the time of lumpectomy surgery or shortly afterwards. IORT accurately targets the tissues that are at highest risk of local recurrence and its efficacy is being tested in an international randomised controlled trial, ‘TARGIT’ that compares it to conventional EBRT. Results published in 2010 suggested equivalent local control and survival with a median follow-up of 3 years and 7 months [7]. Updated 5 year results indicate IORT is still a non-inferior treatment when it is delivered concurrently during lumpectomy but rates of local recurrence when given as a second separate procedure are 3.7% higher than EBRT in the IORT arm (p = 0.069), making this a less preferred option, although survival was equivalent irrespective of the timing of delivery [8]. Potential benefits of IORT include increased convenience, decreased time away from home for country patients and the elderly and decreased time away from family and work for all women. The TARGIT trial toxicity results confirm a different spectrum of toxicities to EBRT including significantly fewer skin toxicities but a higher risk of post-operative seromas [7]. Cosmesis results suggest a better outcome with IORT [9]. The first survival analysis of the TARGIT trial has also shown a significantly lower number of breast cancer related deaths for women receiving IORT (1.4% Vs. 3.5%) [8].

With 5 year data now showing that IORT as a second separate procedure gives lower rates of local control than EBRT it will be important to determine whether women and their clinicians are prepared to accept this difference. Clinicians have an important role in helping patients make decisions about adjuvant radiotherapy following breast conserving surgery. Studies have shown that most cancer patients prefer to make decisions together with their doctor, or even let their doctors decide on their behalf [10,11]. There is limited understanding of clinician attitudes and preferences surrounding radiotherapy choices.

The purpose of this observational study was to identify what maximum increase in risk of local recurrence, if any, health professionals working with breast cancer patients would accept in order to have IORT instead of EBRT for themselves, in a hypothetical setting. The study also aimed to investigate potential socio-demographic and role factors that may impact on the level of health professional acceptance of IORT and whether there was a difference between the responses collected in 2004 and 2011, a period in which experience with IORT had developed. This is the first comprehensive investigation of health professional preferences in a radiotherapy setting and will supplement patient preference data currently being collected as part of the TARGIT Trial.

## Methods

In 2004 and again in 2011 over 200 medical and non-medical health professionals working with breast cancer patients in hospitals with specialist breast units in Western Australia were invited by mail to complete a health professional preference questionnaire. The sampling frames for each year were not available as predefined lists, hence were created by the investigators using knowledge of existing colleagues in the field of breast cancer research and communication with managers of large professional groups such as nurses, radiation therapists, and research data managers. Invitees received a participant information sheet, validated preference questionnaire and reply-paid envelope [12]. The self-administered anonymous preference questionnaire included three pages of written information covering the purpose of the study, a description of the two treatments being compared, two hypothetical preference scenarios, and instructions on how to complete the preference questions. The study had Human Research Ethics Committee approval and return of the questionnaires was accepted as indicating consent. Given the anonymity of the questionnaires, non-responders could not be reminded to return their questionnaires.

The template for the preference questionnaire was developed and validated in previous studies for adjuvant chemotherapy [12,13]. The questionnaire used the trade-off method to determine the maximum increase in risk of local recurrence of EBC that health professionals judged acceptable in order to choose IORT over EBRT [12,14]–[16]. “Trade-off” methodology is a validated and accepted method of determining patient preferences [15]. The trade-off method requires the respondents to consider the positive and negative effects of a treatment together with the probabilities of these effects. In this way, the trade-off method can be used to elicit preferences for a specific treatment.

The questionnaire consisted of two preference questions using different hypothetical baseline risks of local recurrence in the setting of EBRT, socio-demographic questions and six feedback questions about the questionnaire itself. The preference questions asked health professionals to choose either the baseline risk of recurrence of EBRT or an equivalent or higher risk of recurrence in order to have the more convenient, single dose of IORT instead of EBRT.

The preference questions in 2004 had hypothetical baseline risks of recurrence following EBRT set at 6% and 9% (based on local recurrence evidence found in the literature at the time) and were increased for the IORT scenarios by increments of 1% such that the maximum proposed increase in risk to accept IORT over EBRT could be an additional 5% risk of local recurrence [4,17,18]. In 2011 the baseline risks of recurrence were modified to 3% and 6% due to updated reports showing lower rates of recurrence in low-risk early breast cancer patients [6,7]. The risks were once again increased by an increment of 1%, but the maximum proposed increase in risk to have IORT used was 6% (not 5% as in 2004) due to the lower baselines being used. In the two hypothetical scenarios, local recurrence was described as not impacting on overall survival.

Statistical methods included Cronbach’s Alpha to identify whether responses between the 6% and 9% baseline questions in 2004 were interchangeable and likewise with the 3% and 6% baseline questions in 2011. Further analysis was limited to the 6% baseline for each time point to maintain comparability. Health professional acceptability of IORT was categorized into four groups; not acceptable at all (‘*never*’); acceptable if risk is ‘*equivalent*’ to EBRT; acceptable if risk is 1-3% higher than EBRT (‘*low risk*’); and acceptable if risk is 4-6% higher than EBRT (‘*high risk*’).

Results from previous patient and clinician preference studies were used to select variables to include in univariable and multiple Poisson regression analysis to identify if any significant drivers of preferences could be found within the demographic data collected [12,14,19]. Chi^2^ was used to investigate if any significant differences existed between the two time points, professional role groups and different levels of familiarity with the two treatments. Results were summarized by histograms and a chart of cumulative proportions. Double-data-entry was performed on all fields for quality assurance purposes and all statistical analyses were performed using IBM SPSS Statistics version 21 for Windows.

## Results

Questionnaires were sent out to approximately 200 and 317 health professionals in 2004 and 2011 respectively. Numbers are approximate because some centres requested bulk mail outs of questionnaires to groups such as ward nurses and clinical trial units, rather than disclosing individual names for separate postage. It is therefore unknown how many of the questionnaires mailed out in bulk were actually received by a health professional as their distribution was performed by the relevant unit managers at each site. As the number of centres involved in the use of IORT had grown between 2004 and 2011, an additional hospital was included in 2011, and cancer ward nurses at two hospitals who were not targeted in 2004 were also invited in 2011, leading to a higher number of questionnaires sent out in that year.

In 2004, responses were received from 90 health professionals (45% return rate) and in 2011 the return rate was 35% with 110 responses received. Of those received, 80 and 92 were included for analysis in 2004 and 2011 respectively. Exclusion from analysis occurred if a) participants worked less than 5% with breast cancer patients (n = 24, 12%) b) participants had experienced radiotherapy themselves (n = 1, 0.5%) or c) if responses to the preference questions were indeterminate (n = 3, 1.5%). Response rate data is summarized in Table 1.

**Table 1:** Questionnaire response rates in 2004 and 2011

	**2004**	**2011**
Sent out (approximate)	200	317
Received (response rate)	90 (45%)	110 (35%)
Excluded because <5% time worked with Br Ca	9	15
Excluded because had XRT	0	1
Excluded because of invalid answers	1	2
Total number excluded	10 (11%)	18 (16%)
Number analysed	80 89% of those received40% of those sent out	9284% of those received29% of those sent out

Comparison of the 6% and 9% baseline questions in 2004 revealed a significant agreement on Cronbach’s Alpha (0.963, p = <0.0001) as did the comparison of the 3% and 6% baseline questions in 2011 (Cronbach’s Alpha 0.880, p = <0.0001). As a result, all subsequent analyses of preference responses focused only on the 6% baseline question from each year group.

Table 2 summarizes the descriptive characteristics of the 2004 and 2011 participants. Demographics in the two year groups were similar, with the majority of participants being female, having partners and dependents, and having had a friend or relative who had experienced radiotherapy. Differences between the two time points included the number of radiation therapists declining from 26 down to 16 (32% vs. 17%) and an increase in nurses from 29 to 45 (36% vs. 49%) and allied health professionals from 3 to 9 (4% vs. 10%) participating in the study. Allied health professionals included data managers (n = 2 and 5) and psychologists (n = 1 and 4).

**Table 2:** Descriptive characteristics of health professionals in 2004 and 2011

**Characteristic**	**2004 (%) n = 80**	**2011 (%) n = 92**
Female	61 (76)	80 (87)
Median age, years (range)	43.5 (24–63)	45.5 (21–67)
Role group		
Medical	22 (28)	22 (24)
Radiation therapist	26 (32)	16 (17)
Nurse	29 (36)	45 (49)
Allied health	3 (4)	9 (10)
Time dedicated to working with breast cancer (range*)	5-100	5-100
25th Percentile	20	10
50th Percentile	30	30
75th Percentile	67	60
Marital status (has partner)	61 (76)	67 (73)
Has children	54 (68)	58 (63)
Has children <15 yrs	27 (34)	30 (33)
Has dependents	51 (64)	54 (59)
Has relative/friend who died from cancer	63 (79)	81 (88)
Has relative/friend who has had radiotherapy	55 (69)	69 (75)
Ever considered radiotherapy for themselves	2 (2.5)	2 (2.2)
Familiarity with EBRT		
Never heard of it	1 (1.3)	2 (2.2)
Read or heard about it	8 (10)	18 (20)
Supervised patients having it	71 (89)	72 (78)
Familiarity with IORT		
Never heard of it	7 (9)	12 (13)
Read or heard about it	36 (45)	38 (41)
Supervised patients having it	37 (46)	42 (46)

*Note that responses less than 5% were excluded from analysis as suggestive of insufficient experience with breast cancer patients.

The proportion of medical health professionals across the two time points was similar, representing 28% and 24% of the responses in 2004 and 2011 respectively. In 2004 and 2011, the medical group comprised of 7 and 9 surgeons, 6 and 3 medical oncologists, 8 and 6 radiation oncologists, 1 and 2 breast physicians and one radiologist in 2011. One registrar with no specified specialty also responded in 2011. Overall exploration of gender across role groups revealed a p-value approaching significance (Fisher’s Exact Chi^2^ p 0.052) with a decline in number of male medical health professionals (15 vs. 9) and a reciprocal increase in number of female medical health professionals (7 vs. 13) from 2004 to 2011 (Fisher’s Exact Chi^2^ p 0.065).

Despite a larger sample size in 2011, familiarity with either EBRT or IORT did not yield any significant differences between 2004 and 2011. In 2004 and 2011, EBRT had been supervised by 89% then 78%, and had been read or heard about by 10% then 20% respectively. Likewise familiarity with IORT showed little change, with 9% then 13% having never heard or read about it, and a stable number of those having supervised it or read/heard about it.

**Figure 1:**
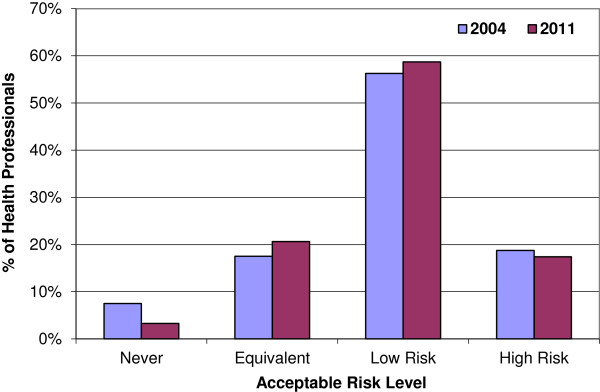
Health professional preferences.

**Figure 2:**
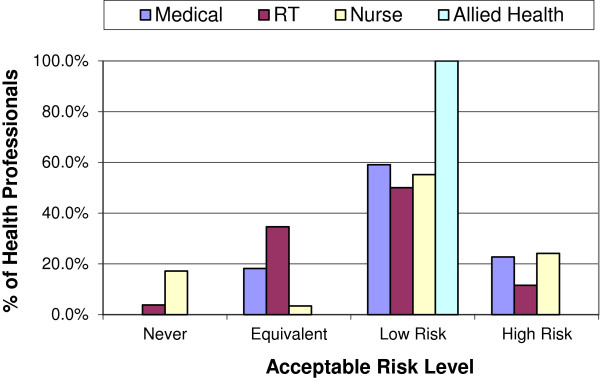
2004 Preferences by role group.

**Figure 3:**
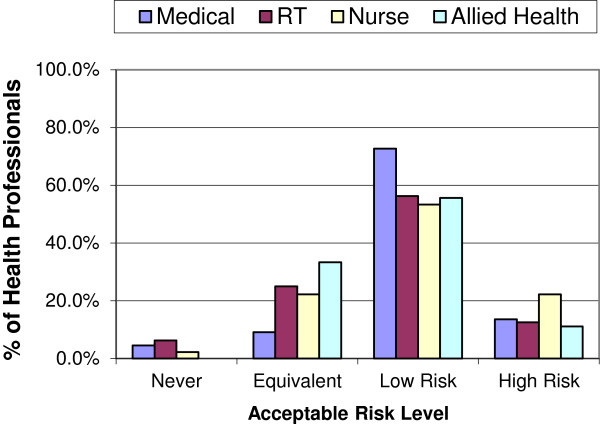
2011 Preferences by role group.

The actual levels of risk health professionals were willing to accept in order to have IORT in place of EBRT varied considerably (Figure 1). In both 2004 and 2011, most health professionals were willing to accept IORT as an alternative treatment option, with only 7.5% and 3.3% (n = 6 and 3) respectively indicating they wouldn’t have IORT at all (‘never’). If it offered an ‘equivalent’ risk of recurrence, 18% and 21% (n = 14 and 19) would have it, with a similar proportion accepting IORT if it offered a 4-6% (‘high’) increase in local recurrence (19% and 17% with n = 15 and 16). The majority of health professionals would accept IORT if it offered a 1-3% (‘low’) increase in local recurrence (56% and 59% with n = 45 and 54). Figures 2 and 3 display the similarity of responses received across role groups in 2004 and 2011 respectively.

**Figure 4:**
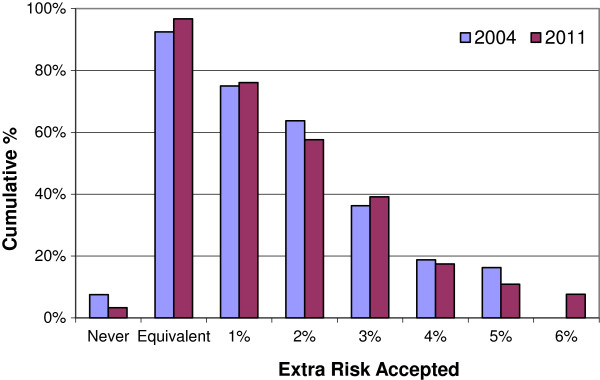
**Cumulative proportions of health professionals accepting IORT.** Note: In 2004, the maximum increase in risk presented was 5% hence there is no 2004 value for the 6% increase in risk.

Figure 4, showing the cumulative increase in risk health professionals are willing to accept in order to have IORT in place of EBRT, further illustrates the similarity between the two time points. This graph suggests that approximately 60% of health professionals would accept IORT if it increased the risk of local recurrence by 2% and approximately 40% would accept it at an increased risk of 3%.

Chi-squared analysis of preference category by role group revealed the only significant difference between the two time points was in the nursing role group (Fisher’s exact p value = 0.026). In 2004, 5 (17.2%) nurses would not have IORT at all compared to 1 (2.5%) in 2011 and 1 (3.4%) would have it only if it offered an equivalent risk of LR in 2004, compared to 10 (22.2%) in 2011. The proportion of nurses accepting IORT with a low or high extra risk did not change between the two time points (55% vs. 53% for low risk and 24% vs. 22% for high risk). Significant differences were not found in any other role group, as illustrated in Table 3.

**Table 3:** Significant preference category changes by Nurses

**Preference**	**2004**	**2011**
Would not have IORT at all	17%	2%
Would have IORT only if equivalent to EBRT	3%	22%

The only significant difference found between the two time points was in the nursing group (Fishers Exact Chi^2^ p = 0.026 ). Differences were not found in the medical group (p = 0.47), radiation therapist group (p = 0.95) or the allied health group (p = 0.61).

Univariable and multiple Poisson regression analyses did not identify any significant drivers of preferences in either year group (Table 4).

**Table 4:** Potential drivers of preference explored with univariable analysis

**Characteristic**	**Univariable *P***-**value**		
Year	2004	2011	Combined
	n = 80	n = 92	n = 172
Gender*	0.29	0.85	0.51
Age*	0.61	0.60	0.47
Marital status*	0.38	0.68	0.37
Has dependents*	0.74	0.38	0.38
Has children younger than 15*	0.82	0.19	0.26
Friend or relative died of cancer*	0.46	0.65	0.38
Role group	0.84	0.95	0.79
Medical vs. non-medical role group*	0.46	0.84	0.52
IORT Familiarity	0.87	0.98	0.94
% time working with breast cancer patients*	0.10	0.59	0.14
Participation year	-		0.85

*Overall multivariable Poisson Regression analysis included these variables yielding an insignificant p-value of 0.685. In 2004 p = 0.850 and in 2011 p = 0.904.

Feedback from health professionals in 2004 and 2011 revealed 84% found the questions clear, few thought answering the questions was hard (10% and 15%), or that the questions were hard to understand (19% and 14%) or stressful (6% and 5%), and most were glad that they took part in the study (69% and 77%). Only 3.8% and 7.6% found the questions both hard to understand and hard to answer in 2004 and 2011 respectively. The median time for completion was 9 minutes (range 2–30) in 2004 and 8 minutes in 2011 (range 1–30).

## Discussion

The few published studies that have reviewed patient preferences with regards to adjuvant radiotherapy and risk of local recurrence have been focused on the trade-off between either adjuvant radiotherapy and mastectomy, or radiotherapy vs. no radiotherapy [20]–[22]. Results of one American study conducted in the late 1990s strongly suggested that the fear of a local recurrence and an actual local recurrence leading to mastectomy have such a negative impact on quality of life that patients are willing to accept the risks and inconvenience of radiation therapy to avoid them, even if there is no benefit to survival [21]. A similar Canadian study also concluded that patients are unwilling to accept even small increases in local recurrence risk to avoid radiotherapy, and that clinicians underestimate the fear that patients have for local recurrence and their desire to use all available treatment options for small gains [22]. Investigation into health professional preferences for chemotherapy also in the 1990s found that health professionals require larger benefits of treatment to make chemotherapy worthwhile compared to patients [23,24].

The historical studies described above make it clear that the value of adjuvant treatment after conservative surgery is dependent on how women feel about the trade-off between the fear and consequences of a local recurrence vs. the toxicity and inconvenience of treatment, and that patients appear to place significant value on the local control of their cancers in terms of their overall quality of life. Health care professionals' attitudes and preferences may determine what treatment options they discuss with patients, and these may differ significantly to the attitudes and preferences of their patients. This may be particularly important when introducing new treatments or technologies where quality of life issues are significant and there is no survival advantage.

In this study, we aimed to understand what determined health care professionals’ preferences for IORT or EBRT in early breast cancer. Despite the absence of any significant determinants of preference, the 2011 data shows a general acceptance of IORT as an alternative treatment to EBRT across all surveyed role groups such that only 3.3% would not accept it all, 80% would have it if it offered either an equivalent or low (1-3%) increase in risk and 17% would have it even if it posed a 4-6% increase in local recurrence. The lack of any identified determinants of preference was unexpected given findings from previous preference studies showing at least one significant driver of preference. A study of lung cancer health professionals found that having a partner or having dependent children was associated with judging smaller survival benefits of chemotherapy sufficient [12]. Preference studies on patient groups have similarly found having dependents at home is a significant determinant of preference in a chemotherapy setting [14,19]. There are no published preference studies for health professionals in radiotherapy, however the only two patient preference radiotherapy studies available also did not find any significant determinants of preference [21,25]. Of note, and similar to the present study, neither of these studies was assessing preferences in the context of survival differences. It is possible that the socio-demographic determinants of preferences such as having a partner or dependent children come into effect when a survival difference is considered, but are less important when survival is not the outcome being considered. Furthermore, factors such as older age, employment and rural location may be hypothesized as important determinants of preferences for patients considering IORT, but could not be included in the health professional study as by definition the participants were both employed and accessible to the healthcare sites as well as working age. IORT can be delivered during the same procedure as tumour removal, or as a separate procedure some time later, however updated results of the TARGIT study have shown the preferred approach is to offer IORT at the same time as tumour removal because the differences in risk of local recurrence compared to EBRT were found to be 1% (p = 0.31) for during initial surgery and 3.7% (p = 0.069) when performed as a separate procedure [8]. Qualitative data collected during this study suggests that preferences for IORT may be influenced by the timing of the procedure, as a separate procedure would require an additional general anaesthetic. The timing of the procedure in addition to personal characteristics independent of socio-demographics may also need to be considered in future studies of health professional preferences involving radiation therapy for early breast cancer, including examination of psychological factors such as risk profiling, and body image.

Although designed as a simple observational cross-sectional study, this study has several potential limitations. The study sample size was restricted by the number of health professionals working with breast cancer patients in Western Australia at the two time points. The devised sampling frames were representative of the majority of health professionals working in breast cancer care in Western Australia in both 2004 and 2011 however they may have under-represented allied health staff such as psychologists, social workers, physiotherapists and palliative care physicians as they made up less than 5% of the sampling frame at both time points. Representative percentages of data managers (9% and 4%), radiation oncologists (7% and 5%), medical oncologists (5% and 4%), breast surgeons (9%, 4%), nurses (39% and 45%), radiation therapists (21% and 20%) and radiologists (5% and 10%) were invited in 2004 and 2011 respectively.

Observed response rates were low which is common in surveys of health professionals, particularly if they are anonymous [26] however the final number of participants in each year group is similar to several relevant published preference studies [19,21,27]. The reduced response rate in 2011 is possibly due to the reluctance of health professionals to complete the questionnaire again if they recalled completing it in 2004, despite the information sheet indicating the investigators wanted them to complete it again because “more is known about IORT now, and it is more likely that you have been involved in the care of patients post IORT now than when you first completed the questionnaire six years ago”. The literature suggests that when surveying health professionals, responder bias is modest, and less important than in surveying patient or consumer groups [28]. However, selection bias may exist due to targeting only health professionals known to be working in the field in one of four major hospitals in Western Australia. The lower IORT familiarity scores in 2011 are possibly a direct consequence of increasing the number of people asked to participate, particularly the nursing group, as the use of IORT had remained relatively stable in clinical practice between 2004 and 2011.

A further potential limitation is that because the focus of the hypothetical scenario in this study was local recurrence, we indicated that survival was exactly the same for both treatments. This study did not measure the opinions of health professionals in regards to this forced assumption of equivalent survival. Updated results of the full TARGIT trial cohort with a median follow-up of 2.5 years have now shown that breast cancer mortality is not different between the two treatments, although significantly fewer non-breast cancer deaths were reported for EBRT [8]. Longer term follow-up is needed to confirm these findings, as the number of events is still low. Despite this recent finding, given the 15-year survival data published in 2005 suggests that for every 4 local recurrences avoided, one life is saved, future health professional preference studies of radiotherapy should incorporate this information [6]. Further to this, we updated the baseline recurrence rates in 2011 from 6% and 9% (used in 2004) to 3% and 6% to be in line with the emerging literature reporting lower local recurrence rates for early, low risk breast cancer patients treated with breast conserving surgery. As we have reported above, the change in baselines didn’t appear to affect this study however, as we used the 6% baselines from both years and analysis showed there wasn’t any significant difference in the way respondents answered the lower and upper baseline questions within each year group. In retrospect, the reduced baselines were appropriate given the updated TARGIT results reporting recurrence rates between 1.1% and 5.4% [8].

There is much patient preference literature available for chemotherapy decisions, however patient preference research for breast radiation treatment is limited and outdated [21]. Specific research on patient preferences for IORT is therefore underway [25,29] and is an important area to consider in anticipation of IORT becoming a standard treatment option. With the updated results of the TARGIT trial showing IORT offers a lower rate of local control than EBRT when offered as a second separate procedure, it will also be important to determine whether patients are prepared to accept this difference. With 17% of health professionals surveyed in 2011 hypothetically accepting an increased risk as high as 4-6%, it is critical to understand which patients have similar preference profiles. Patient preferences should be incorporated in treatment decisions especially when life expectancy varies little and quality of life considerations are prominent. In order to inform such decisions, estimates of the maximum difference in tumour control rates that would be acceptable by patients are also needed.

This study has provided unique information about the preferences of health professionals working with breast cancer patients in regards to IORT and EBRT for early breast cancer. The high acceptance of the questionnaire and brief time required to complete it demonstrates that health care provider preference studies can be implemented with minimal inconvenience to participants and may supplement information on patient preferences where treatment choices are available. This study has shown that determining the preference of health care professionals for one treatment over another is a complex and individual process that cannot be generalized to any particular professional role or socio-demographic characteristic. This data will supplement the clinical results of the TARGIT trial and help clinicians become aware of their own preferences for adjuvant radiotherapy and therefore help patients make well informed and unbiased treatment related decisions should IORT become available as a standard treatment option in the future.

## Conclusions

The majority of Western Australian health professionals working with breast cancer patients surveyed in 2004 and 2011 accepted IORT as an alternative treatment option to EBRT for early breast cancer. Only 3% of health professionals surveyed in 2011 indicated they would not have IORT at all, and 76% would accept it even if it presented an increase in risk of recurrence. This outcome was relatively consistent over two time points spanning 7 years despite the duration of use of IORT and the publishing of the early clinical results of IORT in 2010. These results need to be compared with preferences elicited from patient groups, and further investigation into what drives preferences in health professionals and how this influences recommendations to patients is warranted.

## Competing interests

The authors declare that they have no competing interest.

## Authors’ contributions

TC made substantial contribution to the conception, design and implementation of the study including acquisition of data, performed the statistical analysis, interpreted the data, drafted and revised the manuscript. DJ conceived the study and participated in its design and revised the manuscript. AN made substantial contribution to the conception and design of the study, supervised the implementation of the study and critically revised the manuscript. CS participated in the design and implementation of the study and revised the manuscript. MB supervised the statistical analysis and revised the manuscript. All authors read and approved the final manuscript.

## Authors’ information

TC, DJ, CS, and MB are members of the TARGIT Trial International Steering Committee.
